# Repeatability and reproducibility of quantitative ultrasound cervical measurements in women at risk for preterm birth

**DOI:** 10.1007/s44352-025-00020-3

**Published:** 2025-11-19

**Authors:** Michelle Villegas-Downs, Ziyi Gao, Huizhu Jia, Aiguo Han, Barbara L. McFarlin, William D. O’Brien, Douglas G. Simpson

**Affiliations:** 1 Department of Human Development Nursing Science, UIC College of Nursing, University of Illinois Chicago, 845 S. Damen Ave, Chicago, IL 60612 USA; 2 Department of Statistics, University of Illinois Urbana-Champaign, 725 S. Wright Street, Champaign, IL 61820 USA; 3 Department of Biomedical Engineering and Mechanics, Virginia Polytechnic Institute and State University, 325 Stanger Street, Blacksburg, VA 24061 USA; 4 Bioacoustics Research Laboratory, Department of Electrical and Computer Engineering, University of Illinois at Urbana-Champaign, 306 N. Wright Street, Urbana, IL 61801 USA

**Keywords:** Cervix, Intraclass correlation, Linear mixed model, Preterm birth biomarkers, QUS imaging

## Abstract

**Purpose:**

Transvaginal Quantitative Ultrasound (QUS) has the potential to enhance preterm birth risk monitoring during pregnancy but evidence on the reliability of commonly used QUS parameters in vivo is lacking. This study assesses intra-sonographer repeatability and inter-sonographer reproducibility of six different QUS measurements of the human cervix during pregnancy: Attenuation Coefficient (AC), Lizzi Feleppa (LF) Intercept, Midband and Slope, and Envelope Kappa and Mu.

**Methods:**

This prospective study was approved by the institutional review board at the University of Illinois, Chicago. Informed consent was obtained from all participants, who were selected from pregnant women enrolled in the single-center study, “QUS Technology for Identifying At-Risk Women for Spontaneous Preterm Birth.” They received a standard clinical transvaginal ultrasound scan followed by two research scans at 20 ± 2 and 24 ± 2 weeks of gestation. During one or both research scans, they underwent two independent examinations (same-sonographer or cross-sonographer). QUS measurements were computed from ultrasound radiofrequency (RF) data. Variation attributable to transducers, phantoms, and sonographers was evaluated by linear mixed model analysis. QUS parameters were averaged over four acquisitions from each examination. Repeatability and reproducibility were evaluated using the coefficient of variation (CoV), intraclass correlation coefficient (ICC), and Bland-Altman analysis.

**Results:**

Eighty-one participants (mean age, 27.12 years ± 5.81) were recruited for a total of 82 examination pairs, yielding 36 intra-sonographer and 46 inter-sonographer pairs of examinations. Transducer and reference phantom variances were not statistically significant (*p* > 0.05). AC, LF Midband, LF Slope, Kappa, and Mu displayed moderate re-examination (intra-sonographer) repeatability (CoV: 11.9%-12.9%, ICC: 0.62–0.69). LF Intercept had poor repeatability (CoV: 6.9%, ICC: 0.38). AC and LF Midband also displayed moderate inter-sonographer reproducibility (CoV: 10.4%-13.9%, ICC: 0.61–0.63). LF Intercept had marginal reproducibility (CoV: 7.6%, ICC: 0.51). Kappa and mu had poor reproducibility (CoV: 6.9%-14.5%, ICC 0.27–0.38).

**Conclusion:**

Averaged in vivo transvaginal QUS measurements of AC and LF Midband have potential for development as noninvasive clinical measurements with moderate reproducibility for auxiliary monitoring of the progress of pregnancy. The other QUS parameters evaluated in this study require further refinement before they can be recommended for clinical use.

**Supplementary Information:**

The online version contains supplementary material available at 10.1007/s44352-025-00020-3.

## Introduction

Preterm birth (PTB) is a major public health problem. In the United States, one in 10 pregnant women will deliver preterm, resulting in approximately 380,000 PTBs annually [1, 2]. For infants that survive, the effects can be severe, last a lifetime, and cost society $30 billion a year, far more than any significant adult diagnosis [3, 4]. PTB is a syndrome with multiple possible etiologies [5]. Regardless of the underlying etiology or timing of labor onset (preterm or full-term), a final common pathway involving cervical remodeling, with changes in the cervical tissue microstructure, must occur before the initiation of labor. Advances in ultrasound technology have allowed quantitative information to be obtained from medical ultrasound for diagnostic purposes. Quantitative Ultrasound (QUS) shows promise in being able to predict the risk of preterm birth based on tissue properties rather than pregnancy history or symptoms alone [6–9]. Furthermore, QUS has been found to provide effective biomarkers for fatty liver disease [10].

Here, we assess the repeatability and reproducibility (R&R) of several potential QUS parameters for prenatal screening. We use novel QUS features to investigate R&R of QUS biomarkers in vivo, rather than in phantoms or excised tissue, as was done in previous studies [6–9, 11]. R&R serve as two critical indicators of the precision of quantitative imaging biomarkers [12]. Repeatability refers to the consistency of measurement precision when the same procedures and conditions are maintained across repeated trials (known as repeatability conditions). Reproducibility pertains to the consistency of measurement precision under changing conditions across repeated trials (termed reproducibility conditions).

In routine clinical care, transvaginal cervical ultrasound exams are often used to detect a short cervix, a known risk factor for preterm birth [13]. QUS has the potential for routine application during the same measurement process to yield additional biomarker information during pregnancy [14, 15]. Routine use of such measures as biomarkers requires assessment of R&R [16]. Two specific QUS measurements, Attenuation Coefficient (AC) and Backscatter Coefficient (BSC), have been shown to have acceptable levels of R&R between sonographers using reference phantoms [17]. Additional measures considered here (AC, the BSC derived parameters LF Intercept, LF Midband and LF Slope, and Envelope Kappa and Mu) have previously been considered as potential measures for risk assessment of preterm birth.

As the purpose of the present study is to provide a systematic assessment of the R&R of QUS measures that characterize different aspects of the cervical tissue as it evolves during pregnancy, QUS parameters that are used in soft tissues and defined independently of the scanning system were considered. Attenuation coefficient was selected because the cervix undergoes biochemical changes as pregnancy progresses. These changes include collagen remodeling and changes in hydration that affect how ultrasound energy is absorbed and scattered which are then reflected in AC values [8, 18, 19]. Backscatter coefficient (BSC) was selected because the cervix is composed of dense collagen bundles, as pregnancy progresses BSC is able to detect microstructural changes in collagen organization [20]. In the current study, decibel-scale BSC is equivalent to the Lizzi Feleppa Midband. Lizzi Feleppa Slope and Intercept were also included because they can infer information about tissue microstructure and collagen remodeling by capturing how waves scatter off tissue structures, which can provide insights into changes in collagen fiber organization and spacing [21]. Kappa is related to the ratio of coherent to incoherent scattering and Mu is related to the number density of scatterers, both of which may provide information about tissue heterogeneity, which increases as cervical tissue softens and remodels [22, 23].

These QUS biomarkers were selected to provide a non-invasive, well-defined method to potentially characterize cervical tissue properties using transvaginal QUS acquisitions and measurements in vivo. In this prospective study, we hypothesize that QUS cervical measurements provide repeatable and reproducible biomarkers for routine use during pregnancy. Currently, other than cervical length, there is no other routine quantitative assessment available. Establishing the repeatability of QUS measures would enable clinical use as additional biomarkers, potentially improving preterm birth risk assessment.

## Materials and methods

### Study design and population

The study was approved by the University of Illinois, Chicago Institutional Review Board. Written informed consent was provided by each participant before the first research scan. Parental consent and participant assent were obtained for all participants under the age of 18.

**Fig. 1:**
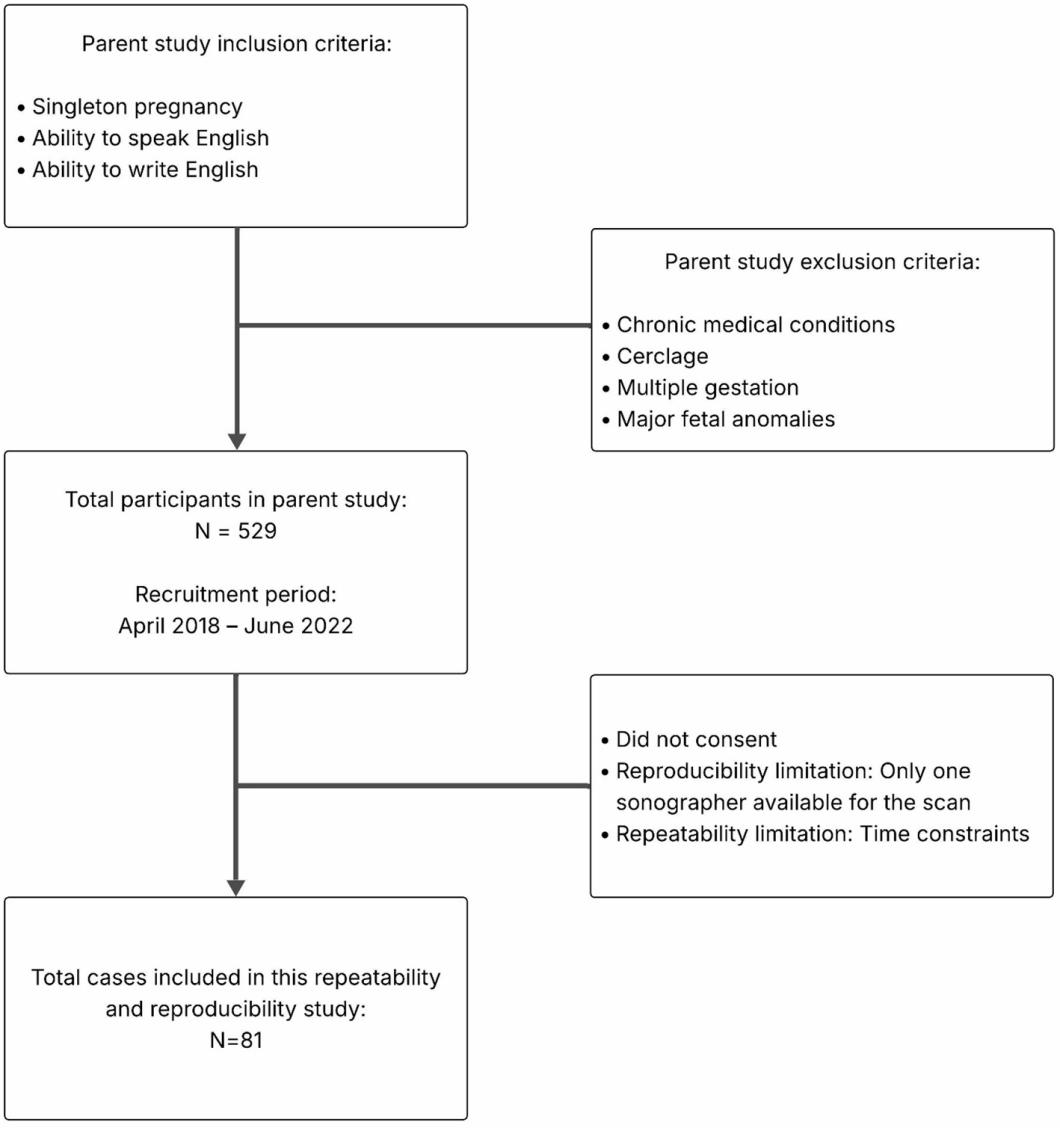
Participant recruitment flow diagram

Participants for this R&R study were selected from a subset of 529 pregnant women who were prospectively enrolled in the single-center study, “QUS Technology for Identifying At-Risk Women for Spontaneous Preterm Birth” [14, 15]. Participants were approached for the R&R portion of the study during scheduled research visits between April 2018 and June 2022. Women from the parent study were included if they were willing to undergo two transvaginal ultrasound exams during a research visit. Not all women enrolled in the parent study were approached for the R&R study. Participation was affected by institutional COVID-19 restrictions that limited building access to one researcher at a time during certain periods, preventing repeat scans involving two sonographers. Additionally, time constraints between appointments restricted our ability to conduct repeat scans with the same sonographer. A formal record of the number of women approached and those that declined were not maintained during recruitment. Thus, selection for the R&R study was non-systematic, rather it was determined by the constraints of conducting the repeated examinations and the time availability of participants. Eligibility criteria for the parent study included participants who were pregnant with a singleton pregnancy and could read and speak English. The study excluded participants with 1) multiple gestation, 2) chronic medical condition(s) (i.e., diabetes, gestational diabetes, hypertension, asthma, autoimmune disorder, preeclampsia), 3) cervical cerclage and/or 4) major fetal anomaly. All participants were drawn from pregnant women whose charts had been pre-screened for eligibility in the parent study. Participants received standard clinical care followed by research screenings consisting of transvaginal scans at 20 ± 2 and 24 ± 2 weeks of gestation. QUS scans were performed on a Siemens Acuson S2000 (Siemens Healthineers, Munich, Germany) by registered diagnostic medical sonographers. Each research screening visit included 11 QUS scans: 10 transvaginal scans taken consecutively and 1 reference phantom (CIRS Inc., Norfolk, VA) scan. The transvaginal scans were calibrated using a reference phantom approach [24]. The custom-made phantoms had a built-in recess (1.5 cm inside diameter, 1.1 cm deep) on its surface that was specifically shaped to fit the curved lens of the Siemens MC9-4 transvaginal probe (1.1 cm radius), allowing for data acquisition across the 176^o^ angle [17]. To ensure accuracy the phantoms were calibrated every 6 months; results of the calibrations did not change during the entire study. Figure 1 shows the participant recruitment flow diagram for the study.

### Sonography data acquisition

Three research sonographers (BTM, 10 years of experience, RDMS; TAP, > 10 years of experience, CCRP; and BLM, > 31 years of experience, RDMS) conducted all the transvaginal scans. All sonographers were trained extensively in the standardized research protocol. Two of the sonographers were proctored and monitored during initial examinations (BTM and TAP) until they demonstrated mastery and consistency in probe placement, image acquisition, and ROI selection. Regular image reviews were conducted to ensure adherence to the protocol throughout data collection. To minimize biological variability, all scans were performed at comparable gestational ages, with participants positioned similarly (supine with a pillow supporting their head on an exam table with stirrups and pelvic tilt) and were asked to empty their bladders before the scan.

Four MC9-4 ultrasonic transducers (measured frequency range: 3.7–6.8 MHz; center frequency 5.25 MHz, denoted TVB, TVG, TVO, and TVR) and three reference phantoms (denoted UIC 3, UIC 4, and UIC 5) were used for the scans. The three phantoms in the current in vivo study are a subset of five phantoms evaluated previously in a phantom-only (ex vivo) R&R study [15]. The reference phantom technique was used to calibrate each set of cervical scans to adjust for system-dependent effects on imaging. For this technique, the phantoms were fabricated with known acoustic parameters. QUS methods in reference phantoms and in vivo using clinical imaging scanners are designed to be reproducible and independent of operator and imaging system factors [24, 25].

For each scan, the participant was instructed to empty their bladder and undress from the waist down. The transvaginal probe was inserted with the focus placed on the cervix at an imaging depth of 5–6 cm, avoiding pressure on the cervix. With each scan, sagittal B-mode images of the cervix where the internal and external *os* (openings of the cervix into the uterus and vagina, respectively) were visible and the raw radiofrequency data were acquired. After each acquisition, there was a cooling off period of 8–15 s. For each of the 10 cervical image acquisitions, the Acuson S2000 settings and probe placement were not changed. The system settings remained the same for the reference phantom acquisition. No specific angle was used, mimicking standard clinical practice for cervical length assessment [11, 26]. Each participant who participated in the R&R study received two independent examinations during a research visit. The protocol remained the same as the standard research scan; however, participants were asked to get dressed, walk down a corridor, and then return for the second scan. The two examinations used the same transducer and phantom. The two examinations were either conducted by the same sonographer to evaluate intra-sonographer variation or by two different sonographers to evaluate inter-sonographer variation.

### QUS data processing

QUS biomarkers were derived from the raw radiofrequency (RF) backscattered signals within the field of interest (FOI) identified by the medical sonographer. The sonographer drew the FOI freehand to capture as much of the homogenous cervical tissue as possible, extending from the external *os* to the internal *os*. The FOI size was not standardized between images. In QUS image processing each FOI was tiled with a regular grid of sub-FOIs of fixed angle width and depth. The calculated QUS measurements included Attenuation Coefficient (AC), Lizzi-Feleppa slope (LF Slope), Lizzi-Feleppa intercept (LF Intercept), Lizzi-Feleppa midband (LF Midband), and envelope statistics Kappa and Mu described in detail previously [14, 15].

In brief, AC, measured using the spectral log difference method, quantifies ultrasound energy loss in the scanned tissue. The backscatter coefficient is a frequency-dependent measure of ultrasound energy reflected from the tissue. LF Intercept, LF Midband, and LF Slope are measures derived from linear regression of decibel scale backscatter coefficients versus frequency and are related to the size and distribution of scatterers within the FOI [15]. Envelope Kappa and Mu are parameter estimates of a homodyned K distribution for the envelope amplitude of the RF data [16, 27]. Because they characterize different physical features of the scanned tissue, it was hypothesized that they could be clinically relevant biomarkers for cervical tissue changes during pregnancy, and these particular biomarkers have been identified previously in QUS characterization of tissue [14–16, 27–29].

**Fig. 2:**
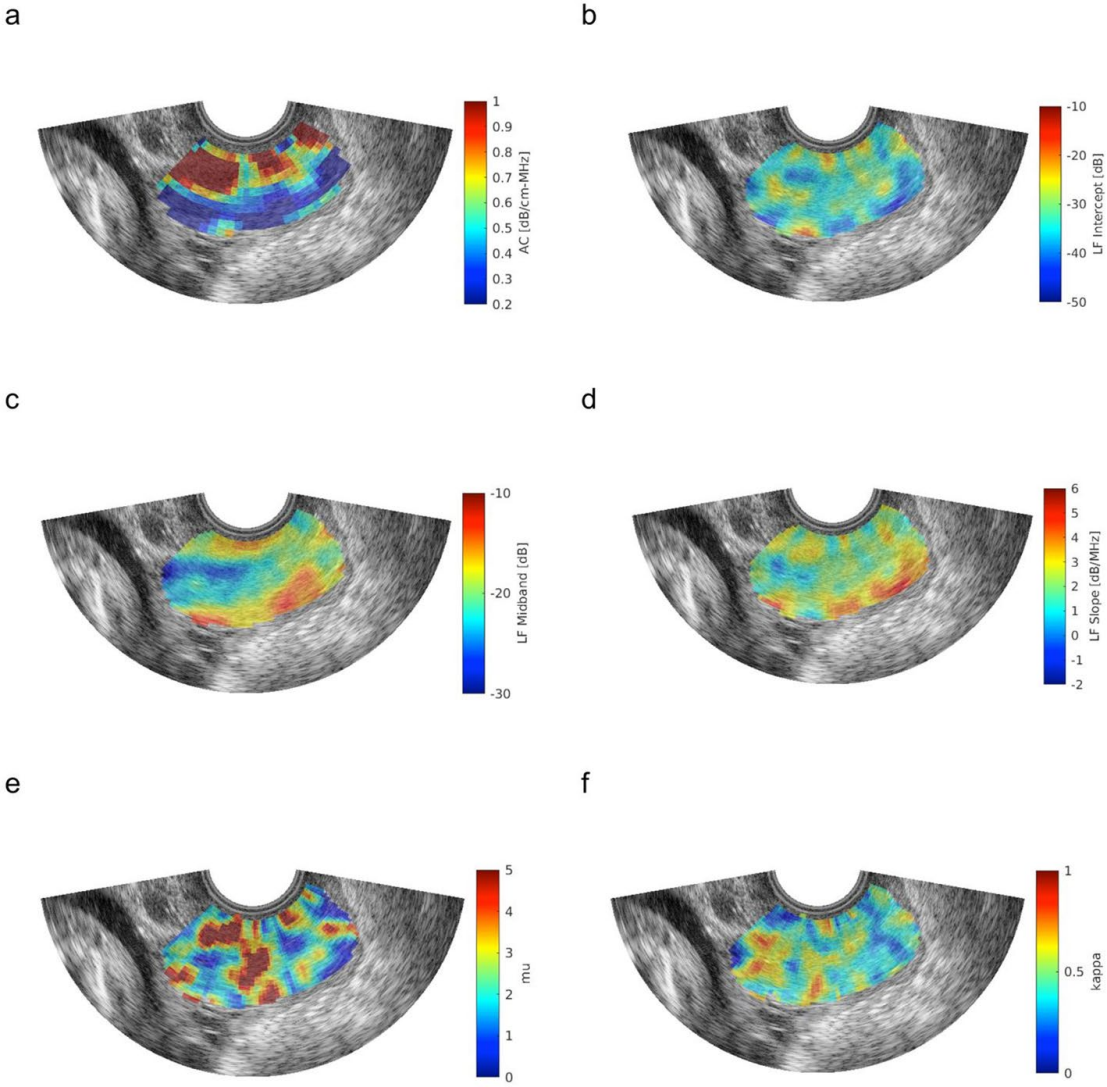
B-mode image of the cervix with FOI and heat map of sub-FOI QUS parameters using Siemens 5–9 MHz vaginal probe, chosen because of its use in clinical practice. Parameters shown are **a** Attenuation Coefficient (dB/ cm-MHz), **b** LF Intercept (dB), **c** LF Midband (dB), **d** LF Slope (dB/MHz), **e** Kappa, and **f** Mu

The six QUS biomarkers were computed from RF ultrasonic data acquisitions using methods detailed previously (AH, with over 10 years of experience). For each acquisition, the mean FOI value for a QUS measurement was obtained by averaging over a grid of sub-FOIs. The FOI and sub-FOI approach is illustrated in Fig. 2 for the six QUS parameters under investigation. The figure shows a cropped B-mode image of the FOI for one scan. The six sub-figures show the sub-FOIs and QUS parameter values within sub-FOIs represented by the color scale to the right of the figure. For each scan, the QUS parameter mean values across sub-FOIs were stored for downstream analysis.

### Statistical analysis

Mean QUS values across sub-FOIs were used as response measures in the statistical analysis. All statistical analyses were performed using R statistical software, version 4.3.2 (R Core Team, Vienna, Austria). Random effects analyses of first acquisition data for all of the QUS measurements were performed using the ‘lme4’ package in R to determine whether transducer, phantom, or visit (research visit 1 versus research visit 2) had statistically significant random effect components of variation. In the absence of statistically significant transducer, phantom, or visit effects, these factors were removed from subsequent analysis. Random effect analysis of multiple acquisition data was conducted on the paired measurements from the two exams for each participant visit in the study including random effects for participant visit, sonographer, and measurement error after averaging over multiple acquisitions (first only, first 2, first 3, etc.). The subset of paired exams with the same sonographer were analyzed separately from the subset with different sonographers. The intra-sonographer repeatability and inter-sonographer reproducibility between the paired exams for all the study participants were assessed via Bland Altman analysis [30, 31] of bias and 95% limits of agreement (LoA) and intraclass correlation (ICC).

ICC values for R&R were derived from the two-way random effects model for the paired exams, with random effects for participants, sonographers (intra- or inter), and error (noise), the repeatability intraclass correlation coefficient (ICC) between the repeated measurements for the same participant is given by

$$\:{ICC}_{Repeat}=\:\frac{{\sigma\:}_{Participant}^{2}}{{\sigma\:}_{Participant}^{2}+\:{\sigma\:}_{e}^{2}},$$

with $\:{\sigma\:}_{Participant}^{2}=$ participant variance and $\:{\sigma\:}_{e}^{2}=\:$measurement error variance. The reproducibility ICC is given by

$$\:{ICC}_{Reprod}=\:\frac{{\sigma\:}_{Participant}^{2}}{{\sigma\:}_{Participant}^{2}+{\sigma\:}_{Inter-sonographer}^{2}+\:{\sigma\:}_{e}^{2}},$$

with $\:{\sigma\:}_{Inter-sonographer}^{2}=$ variance between sonographers. To determine the impact of averaging acquisitions, ICC values and confidence intervals were computed for averaging the first *k* (out of 10) acquisitions for each examination for each of the QUS measurements for *k* = 1, 2, …, 10 to determine the trade-off between acquisition effort and precision gain from averaging. Commonly used benchmark values for ICC are as follows: ICC > 0.9, excellent; ICC > 0.75, good; ICC > 0.5, moderate; ICC < 0.5, poor.

ICC values were computed from the two-way random effects model (single measure) option in the “irr” package in R. Bland Altman analysis of bias and 95% limits of agreement (LoA) was performed using the “blandr” package in R.

## Results

### Participant characteristics

Eighty-one participants were recruited for a total of 82 examination visit pairs (mean age, 27.12 years ± 5.81). Of these, 36 were intra-sonographer pairs and 46 were inter-sonographer pairs of exams conducted under identical circumstances. Table 1 presents numerical summaries of the participant characteristics.

All participants had 10 acquisitions per exam, plus the phantom acquisition. The tenth acquisition for one of the participant exams was found to be of low quality, causing the QUS image analysis to fail. The other 1639 images in the study were processed and included in the data analysis. The one missing observation has no impact on the analysis that averaged over first k acquisitions (k = 1, 2, … 9). It was omitted from the 10-acquisition analysis, resulting in 1.2% missing data for the 10-acquisition analysis only.

**Table 1:** Summary of participant demographics (counts) and characteristics (mean ± standard deviation; range from minimum to maximum)

Characteristics	Results
Number of participants	81
Participant age first clinical visit	27.12 ± 5.81 (Range: 17–40)
Total number of pregnancies (gravidity)	2.78 ± 2.00 (Range: 1–9)
Number of prior preterm births	0.346 ± 0.505 (Range: 0–2)
Self-identified race	
Asian	1
Hispanic	21
Non-Hispanic Black / African American	36
Non-Hispanic White	14
>1 race	4
Other / Declined to answer/No answer	5

**Fig. 3:**
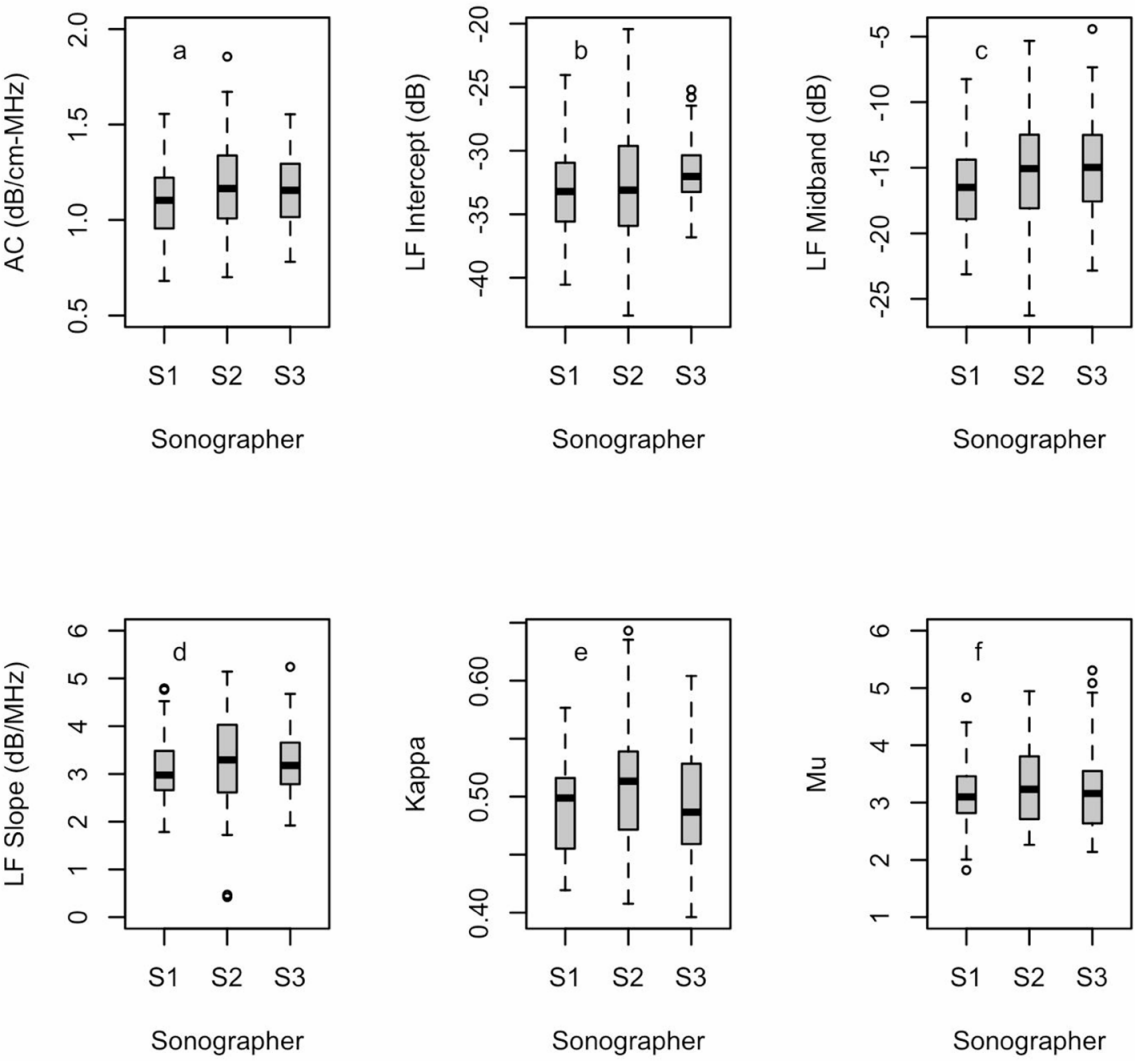
Box plots of participant measurement distributions across sonographers for **a** Attenuation Coefficient (dB/cm-MHz), **b** LF Intercept (dB), **c** LF Midband (dB), **d** LF Slope (dB/MHz), **e** Kappa, and **f** Mu

Figure 3 shows box plots of AC, LF Intercept, LF Midband, LF Slope, Kappa, and Mu for the 3 different sonographers in this study, which provides an overview of the distribution and variability of the measures. Visual inspection of the box plots reveals that the median variation between sonographers is much smaller than the variation of the measured values across participants for each of the six QUS measurements. The distribution of measured values was consistent across sonographers.

### First acquisition components of variability

Table 2 presents a statistical analysis of components of variance for the six QUS measurements using only the first acquisitions. The variations are expressed as random effect standard deviations, and the table provides 95% confidence intervals for each component in parentheses. The values in the table are random-effect standard deviations attributed to each source. The confidence intervals provide a range in which the true standard deviation is expected to lie with 95% certainty. One of the 81 participants contributed two examination visits (one inter-sonographer and one intra-sonographer visit), four weeks apart, to this study. Each of the other 80 participants contributed only one examination visit. For analysis, the 82 examination visits were analyzed as independent paired examinations.

The “Participant” and “Error” sources of variation have the largest effects for all six QUS measurements, and the “transducer” and “Phantom” did not show statistically significant variation. The “transducer” source of variation for the “LF Intercept” and Phantom used for the “LF Slope” is presented as 0, indicating no observed variability due to these factors within the precision of the measurements. Estimated sonographer variation was small compared to residual variation for all measures, and the sonographer variance component was not statistically significant (*p* > 0.05) for any of the QUS measurements.

### Intra- and inter-sonographer variability

**Fig. 4:**
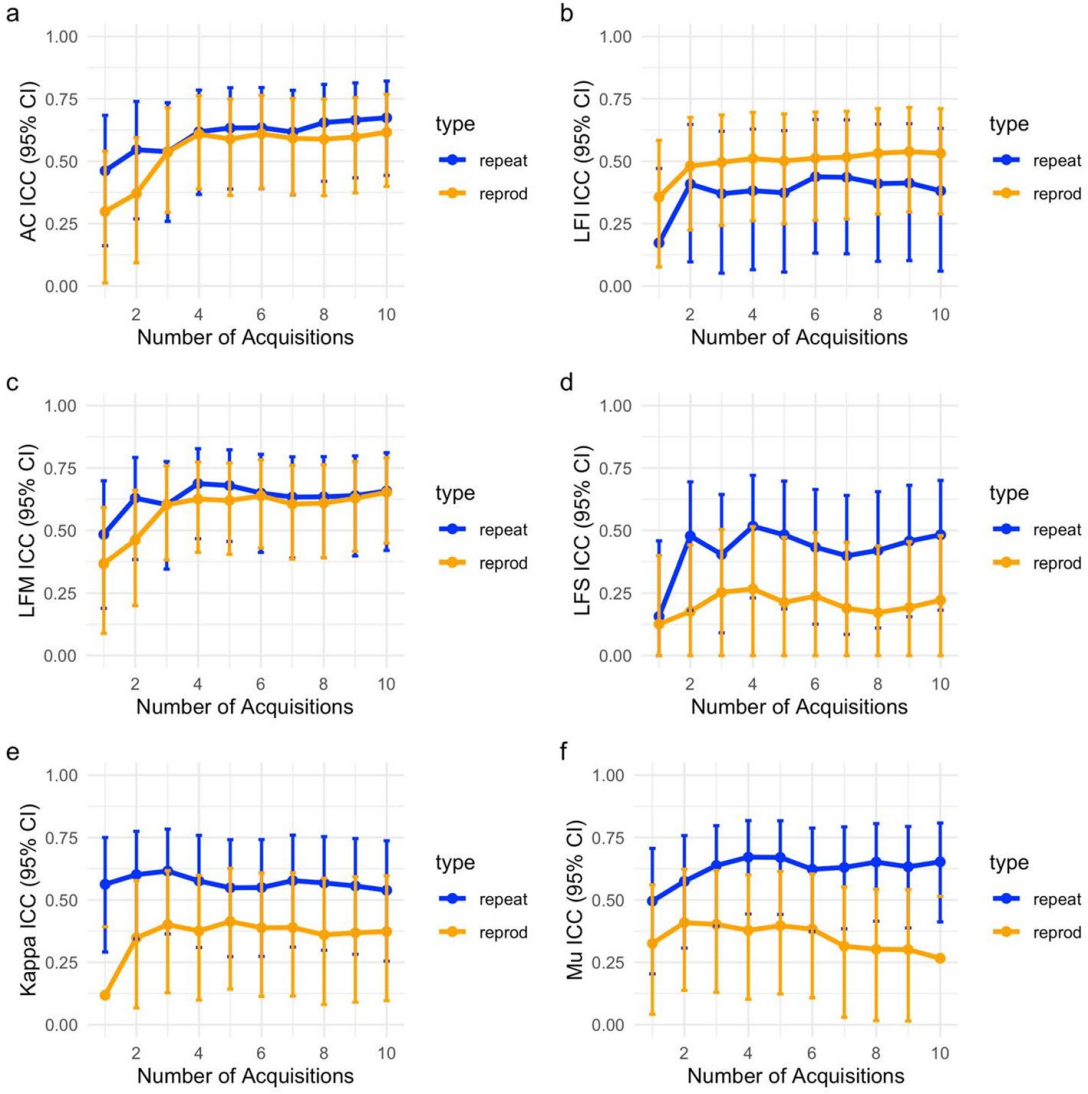
Intra-sonographer repeatability (ICC_Repeat_) and inter-sonographer reproducibility (ICC_Reprod_) versus number of acquisitions: **a** AC, **b** LF Intercept (LFI), **c** LF Midband (LFM), **d** LF Slope, **e** Kappa, and **f** Mu

Given the lack of statistical significance of transducer and phantom effects, a simplified two-way random effects analysis was conducted using the ‘irr’ package in R to estimate intraclass correlation coefficients and compute 95% confidence intervals. Intra-sonographer repeatability ICC and inter-sonographer reproducibility ICC (single measure) were computed after averaging the first k acquisitions from each scan, for k = 1, 2, 3, …, 10. The ICC values and 95% confidence intervals for all size QUS parameters are shown in Fig. 4. For AC and LF Midband, averaging four acquisitions was sufficient to achieve moderate (ICC > 0.50) R&R. LF Intercept had poor repeatability (ICC < 0.50) despite moderate reproducibility for larger numbers of acquisitions. LF Slope, Kappa, and Mu had poor reproducibility (ICC < 0.50) regardless of the number of acquisitions that were averaged.

**Table 2:** First acquisition random effect components of variation for six QUS measurements, expressed as standard deviations with 95% confidence intervals in parentheses

Measurement	Participant	Sonographer	Visit (V1/V2)	Transducer	Phantom	Error
AC (dB/cm-MHz)	0.14 (0.09, 0.18)*	0.03 (0, 0.11)	0.00 (0.00, 0.10)	0.00 (0.00, 0.07)	0.00 (0.00, 0.07)	0.17 (0.15, 0.20)*
LF Intercept (dB)	2.01 (0.96, 2.78)*	0.42 (0, 1.48)	0.61 (0.00, 2.34)	0.00 (0.00, 1.06)	0.00 (0.00, 1.33)	3.08 (2.67, 3.62)*
LF Midband (dB)	2.54 (1.83, 3.26)*	0.44 (0, 1.61)	0.55 (0.00, 2.29)	0.00 (0.00, 1.04)	0.00 (0.00, 1.18)	2.94 (2.56, 3.47)*
LF Slope (dB/MHz)	0.33 (0, 0.51)	0.00 (0, 0.21)	0.00 (0.00, 0.26)	0.00 (0.00, 0.21)	0.00 (0.00, 0.22)	0.77 (0.67, 0.91)*
Envelope Kappa	0.03 (0.01, 0.04)*	0.01 (0, 0.03)	0.00 (0.47, 0.63)	0.00 (0.00, 0.02)	0.00 (0.00, 0.02)	0.04 (0.03, 0.05)*
Envelope Mu	0.44 (0.32, 0.56)*	0.10 (0, 0.32)	0.00 (0.00, 0.26)	0.00 (0.00, 0.20)	0.00 (0.00, 0.22)	0.53 (0.47, 0.63)*

*95% confidence interval excludes zero, therefore the effect is statistically significant (*p* < 0.05).

Table 3 summarizes and expands the repeatability results based on averaging four acquisitions. AC, LF Midband, Mu, and Kappa had repeatability ICC values in the moderate range (> 0.50). LF Slope ICC was close to the threshold between poor and moderate (ICC = 0.52). LF Intercept had poor repeatability ICC (< 0.50). The six measures had coefficients of variation (CoV) ranging from 6.1% to 14.2%.

Table 4 summarizes the reproducibility results based on averaging four acquisitions. AC and LF Midband had the highest ICC for reproducibility, in the moderate range, and coefficients of variation of 13% or lower. LF Intercept, with ICC = 0.51, was near the threshold between poor and moderate, despite a relatively low CoV (6.8%). LF Slope had poor reproducibility ICC and moderately high CoV (20.2%). Kappa and Mu both exhibited poor reproducibility ICC values despite lower CoV values.

Results based on only the first acquisition are provided in the Supplementary Material, Tables S1 and S2. Those single-acquisition results are weaker across all measures than the corresponding results based on four acquisitions presented above.

**Fig. 5:**
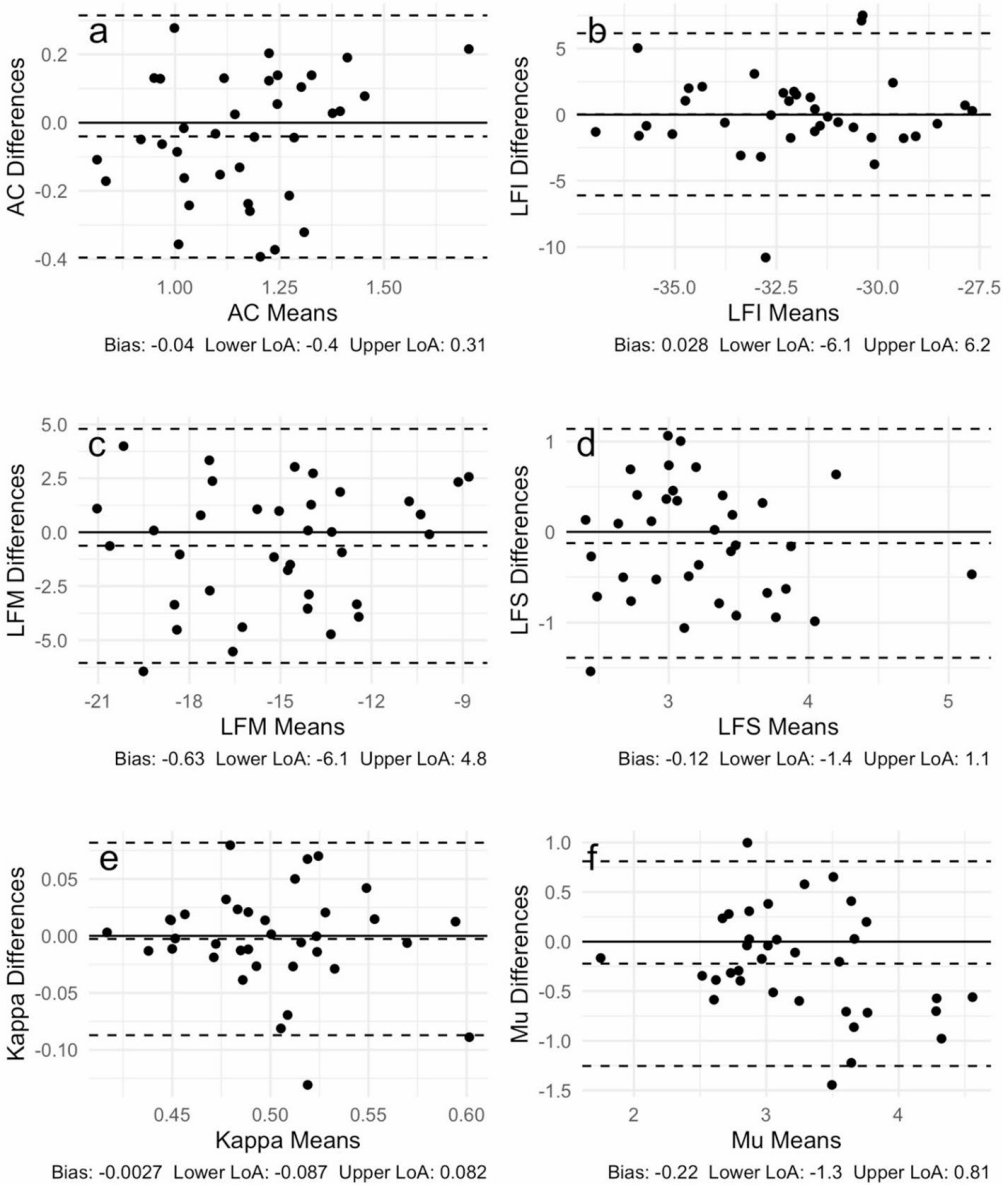
Repeatability Bland-Altman plots based on mean of first 4 acquisitions for **a** AC (dB/cm-MHz), **b** LF Intercept (LFI in dB), **c** LF Midband (LFM in dB), **d** LF Slope (LFS in dB/MHz), **e** Kappa (unitless), and **f** Mu (unitless). Dashed lines show bias and lower and upper LoA (95%); numerical values for bias and lower and upper LoA are displayed below each plot

**Fig. 6:**
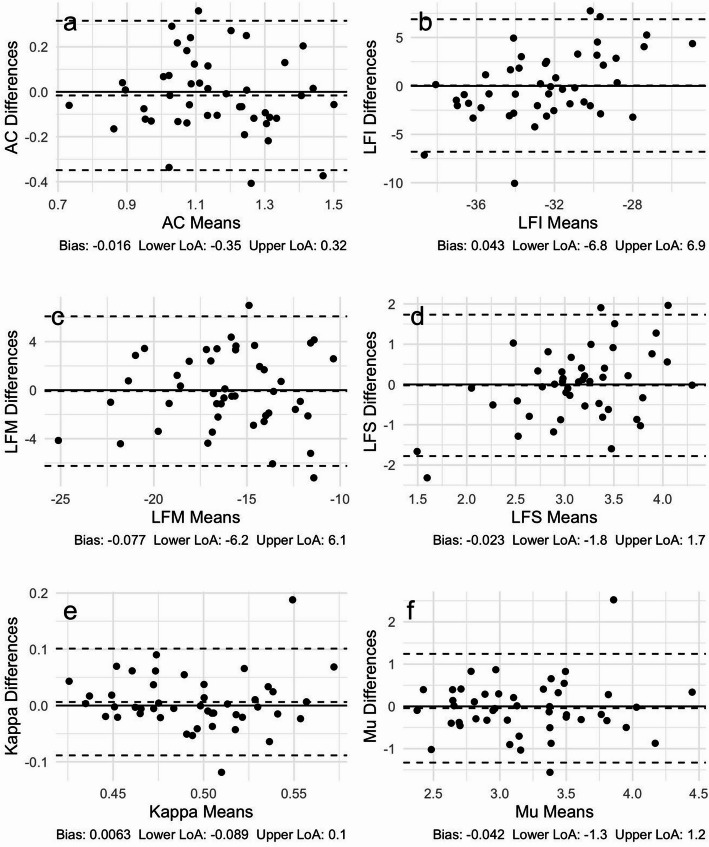
Reproducibility Bland-Altman plots based on mean of first 4 acquisitions for **a** AC (dB/cm-MHz), **b** LF Intercept (LFI in dB), **c** LF Midband (LF in dB), **d** LF Slope (dB/MHz), **e** Kappa (unitless), and **f** Mu (unitless). Dashed lines show bias and lower and upper LoA (95%); numerical values for bias and lower and upper LoA are displayed below each plot

The Bland-Altman plots of paired exam differences versus means are shown in Figs. 5 and 6, where the six plots in each figure correspond to AC, LF Intercept, LF Midband, LF Slope, Kappa and Mu. Figure 5 displays the results for repeatability, whereas Fig. 6 displays the results for reproducibility. The bias and lower and upper limits of agreement (LoA) are included in the caption for each plot. For contrast, the corresponding Bland-Altman plots for single acquisition only are provided in Figures S1–S2 of the Supplementary material.

### Distributional assumptions

Two assumptions of the Bland-Altman analysis are approximate normality and homoscedasticity (constant variance across the range). Normality was assessed via the Shapiro-Wilk test [32] applied to the paired differences within the same-sonographer and cross-sonographer subsets of the data. Results of this analysis are included in Tables S3 and S4 of the Supplementary Material. For the 4-acquistion data, based on the means of the first 4 image acquisitions during each exam, non-normality was detected for LF Intercept same-sonographer data, and for Envelope Kappa and Mu cross-sonographer data. Examination of the corresponding Bland-Altman plots indicates that this may be due to an outlier measurement in each case. Heteroscedasticity was assessed *via* the Breusch-Pagan test [33], applied to the linear regression of paired differences between exams on the corresponding paired means. The results are summarized in Tables S5 and S6 of the Supplementary Material. Heteroscedasticity was detected only for LF Midband in the cross-sonographer data. The statistical significance of this result weak and is no longer significant after Bonferroni adjustment of the p-value for multiple testing.

**Table 3:** Summary of intra-sonographer repeatability results based on the mean of the first four acquisitions during each examination

Measurement	Mean	Bias	Lower LoA	Upper LoA	SD	RC	CoV (%)	ICC
AC (dB/cm-MHz)	1.2	-0.040	-0.40	0.32	0.18	0.36	11.0	0.62
LF Intercept (dB)	-32.1	0.028	-6.1	6.2	3.1	6.1	6.9	0.38
LF Midband (dB)	-15.1	-0.63	-6.1	4.8	2.8	5.4	12.9	0.69
LF Slope (dB/MHz)	3.2	-0.13	-1.4	1.1	0.65	1.3	14.2	0.52
Envelope Kappa	0.50	-0.003	-0.087	0.082	0.043	0.084	6.1	0.58
Envelope Mu	3.2	-0.22	-1.26	0.81	0.53	1.0	11.6	0.67

CoV coefficient of variation (%), ICC intraclass correlation coefficient, LoA limits of agreement, RC repeatability coefficient, SD standard deviation.

**Table 4:** Summary of inter-sonographer reproducibility results based on the mean of the first four acquisitions during each examination

Measurement	Mean	Bias	Lower LoA	Upper LoA	SD	RDC	CoV (%)	ICC
AC (dB/cm-MHz)	1.2	-0.016	-0.35	0.32	0.17	0.33	10.4	0.61
LF Intercept (dB)	-32.4	0.043	-6.8	6.9	3.5	6.8	7.6	0.51
LF Midband (dB)	-16.0	-0.077	-6.2	6.1	3.1	6.2	13.9	0.63
LF Slope (dB/MHz)	3.1	-0.23	-1.8	1.7	0.90	1.8	20.2	0.27
Envelope Kappa	0.50	-0.006	-0.089	0.10	0.048	0.095	6.9	0.38
Envelope Mu	3.2	-0.42	-1.33	1.2	0.66	1.3	14.5	0.38

CoV coefficient of variation (%), ICC intraclass correlation coefficient, LoA limits of agreement, RDC reproducibility coefficient, SD standard deviation.

## Discussion

*Summary of findings*.

The assessment of R&R is essential to validating QUS biomarkers for routine clinical use [12, 16, 34]. In our study, certain measurements of the cervix using QUS during pregnancy demonstrated moderate repeatability under the same conditions (ICC in the range 0.50–0.75) based on averages of 4 acquisitions. These included AC, LF Midband, LF Slope, and Envelope Kappa and Mu. Among these, AC and LF Midband also had reproducibility ICC estimates demonstrating moderate reproducibility (ICC in the range 0.50–0.75). Overall, AC and LF Midband showed the best performance in terms of both R&R. On the other hand, LF Intercept, LF Slope, and Envelope Kappa and Mu each displayed either poor repeatability or poor reproducibility. We note that LF Slope and LF Intercept are inherently more variable than LF Midband, due to the linear regression derivation of these parameters. Envelope kappa and mu displayed non-normality, and further transformations might improve their performance, though it is also possible that the scattering properties they are designed to detect are less well-defined in cervical tissue than are attenuation and backscatter.

Han et al. [35] investigated how inter-sonographer reproducibility was affected by the number of acquisitions used, noting that multiple acquisitions are often collected in practice. Their findings showed that when five acquisitions were averaged per sonographer, the ICC values slightly improved for AC and log BSC, suggesting that more acquisitions improve reliability. Like their findings, in this study, the inter-sonographer and intra-sonographer ICC values were calculated based on the average of four acquisitions, the smallest number of acquisitions required to achieve optimal R&R ICC values. In routine clinical practice, women undergoing transvaginal ultrasound for cervical length monitoring should have a minimum of three measurements taken during the examination [36]. Therefore, obtaining four acquisitions, as was done in this study, would be feasible in clinical practice.

Chen et al. [17] conducted an ex vivo R&R study on transvaginal QUS phantoms for obstetric applications. Investigators reported that AC and log BSC biomarkers measured with transvaginal QUS phantoms using a reference phantom approach were repeatable and reproducible among sonographers, transducer probes, and probe covers. Compared to other studies, their results showed more variability due to the number of factors (sonographers, transducer probes, and probe covers). They hypothesized that similar in vivo R&R studies would have lower R&R due to the additional variability caused by biological tissues. This reflects our findings. While our results showed moderate R&R for AC and LF Midband, the complexity of factors and biological variability resulted in lower ICCs than were observed in the phantom study. Our previous studies [14, 15] have demonstrated that QUS measurements can provide valuable information about cervical tissue microstructure, which helps predict who is at risk for preterm birth. Therefore, even with moderate R&R, QUS measurements of the cervix provide usable, actionable information and may still be sufficient for monitoring preterm birth risk in a clinical setting. However, future studies are needed to determine if training and standardization of protocols can improve R&R values.

The overarching goal of this research is to use innovative QUS technology to identify women at risk for spontaneous preterm birth. Our group has published our acquisition and training protocols [14, 15, 28, 37], which we have tried to align with current standard clinical practices. These protocols have been used consistently in our projects, as standardization is essential for future adoption. Furthermore, our research has focused on evaluating whether QUS biomarkers can be used as complementary features to enhance historical clinical data in predicting spontaneous preterm birth. Future models may consider algorithms that incorporate cervical length measurements to more accurately identify risk.

*Limitations*.

The scope of this study was partly limited by restrictions related to coronavirus disease 2019 (COVID-19). All research activity was suspended from March 2020 until February 2021. After this period, while restrictions were eased, only one sonographer was allowed in the clinic to scan participants for the next year, inhibiting our ability to have two sonographers scan participants for this study. Although the study participants were diverse in terms of race and ethnicity, the relatively small study design did not support for meaningful subgroup comparisons of R&R, which is a potential future direction for research.

The current analysis only considered spatial mean values of the QUS parameters over sub-FOIs to ameliorate the effect of tissue heterogeneity within the FOI. Further work to determine how to incorporate the spatial variation of the sub-FOI measurements into the data processing might improve performance downstream.

Demographic characteristics were included to ensure adequate and realistic variation between participant measurements, but such factors, being confounded with between-participant random effects, did not enter the within-participant assessment of R&R in our analysis. Similarly, biological factors such as cervical mucus and maternal positioning contribute to the random error or noise, but do not cause bias because of the paired exam design.

## Conclusion

These findings support the potential use of AC and LF Midband as biomarkers in mid-pregnancy to monitor the risk of preterm birth and the progression of a normal pregnancy. In addition, the lack of significant differences in measurements between sonographers (*p* > 0.05) further supports the reproducibility of the QUS approach to non-invasive diagnostics. Across three sonographers, four transducers, three phantoms, and 81 pregnant participants, the R&R ICC values for AC and LF Midband were high enough to suggest that the QUS measurements had moderate reproducibility for use as clinical biomarkers during pregnancy.

The findings of this research are important because in our original outcome studies [14, 15], we analyzed one out of ten data acquisitions (scans). If we analyze repeated data acquisitions for each participant visit, there is a potential for improved preterm birth prediction. Until recently the only clinically effective method to determine spontaneous preterm birth risk in the general obstetric population, has been to wait for symptoms of labor [11].

Mid-pregnancy QUS measurements of the cervix can potentially identify women at risk for preterm birth. To advance QUS research and its clinical application, reliable measurements are needed. This study provides evidence that AC and LF Midband measurements exhibit sufficient reproducibility to be used in clinical settings for the routine monitoring of changes in cervical tissue during pregnancy. The other measurements included in this study may require further methodological refinement before proving useful in the clinic, however, as modern machine learning technology advances even weak features might prove useful in diagnostic medicine.

## Supplementary Information

Below is the link to the electronic supplementary material.

Supplementary Material 1

Supplementary Material 2

## Data Availability

Data supporting the findings of this study are included within the manuscript and supplementary information. Additional data may be made available upon reasonable request to qualified researchers, subject to legal and regulatory requirements.
