# Supplementary material for: Repeatability and reproducibility of quantitative ultrasound cervical measurements in women at risk for preterm birth
Source: Discov Imaging. 2025 Nov 19;2(1):16. doi: 10.1007/s44352-025-00020-3 (PMC12627178; doi:10.1007/s44352-025-00020-3)
Supplement: Supplementary file 1 — Supplementary Material 1 [file 44352_2025_20_MOESM1_ESM.docx]

**Supplementary material**

**Table S1:** Shapiro-Wilk test results for normality of Exam 1 – Exam 2 differences based on the first acquisition from each examination. W is the Shapiro-Wilk Statistic: the null hypothesis is that the residuals are normally distributed.

|  | Same Sonographer | | Cross Sonographer | |
| --- | --- | --- | --- | --- |
| Measurement | W | p-value | W | p-value |
| AC | 0.971 | 0.453 | 0.988 | 0.901 |
| LF Intercept | 0.923 | 0.015 | 0.953 | 0.061 |
| LF Midband | 0.982 | 0.812 | 0.988 | 0.921 |
| LF Slope | 0.984 | 0.882 | 0.988 | 0.907 |
| Kappa | 0.928 | 0.022* | 0.954 | 0.068 |
| Mu | 0.983 | 0.837 | 0.928 | 0.007* |

*p < 0.05 indicates statistically significant non-normality.

**Table S2:** Shapiro-Wilk test results for normality of Exam 1 – Exam 2 differences based on the mean of the first 4 acquisitions from each examination. W is the Shapiro-Wilk Statistic: the null hypothesis is that the residuals are normally distributed.

|  | Same-Sonographer | | Cross-Sonographer | |
| --- | --- | --- | --- | --- |
| Measurement | W | p-value | W | p-value |
| AC | 0.965 | 0.311 | 0.973 | 0.359 |
| LF Intercept | 0.901 | 0.004* | 0.974 | 0.394 |
| LF Midband | 0.963 | 0.273 | 0.983 | 0.728 |
| LF Slope | 0.977 | 0.657 | 0.989 | 0.942 |
| Kappa | 0.941 | 0.055 | 0.923 | 0.005* |
| Mu | 0.995 | 1.00 | 0.927 | 0.007* |

*p < 0.05 indicates statistically significant non-normality.

**Table S3:** Breusch-Pagan heteroscedasticity test results for the regression of exam differences on exam means for the first acquisition from each examination. BP is the test statistic value. Under the null hypothesis of homoscedasticity, BP is approximately a chi-square statistic on 1 degree of freedom.

|  | Same Sonographer | | Cross Sonographer | |
| --- | --- | --- | --- | --- |
| Measurement | BP | p-value | BP | p-value |
| AC | 0.015 | 0.904 | 3.10 | 0.078 |
| LF Intercept | 0.003 | 0.956 | 3.36 | 0.067 |
| LF Midband | 0.037 | 0.847 | 4.28 | 0.039* |
| LF Slope | 0.090 | 0.764 | 0.34 | 0.558 |
| Kappa | 0.682 | 0.409 | 0.68 | 0.41 |
| Mu | 0.108 | 0.743 | 1.29 | 0.256 |

*p < 0.05 indicates statistically significant non-normality.

**Table S4:** Breusch-Pagan heteroscedasticity test results for the regression of exam differences on exam means for the mean of the first four acquisitions from each examination. BP is the test statistic value. Under the null hypothesis of homoscedasticity, BP is approximately a chi-square statistic on 1 degree of freedom.

|  | Same-Sonographer | | Cross-Sonographer | |
| --- | --- | --- | --- | --- |
| Measurement | BP | p-value | BP | p-value |
| AC | 0.00 | 0.993 | 0.348 | 0.555 |
| LF Intercept | 0.0012 | 0.972 | 0.073 | 0.787 |
| LF Midband | 3.43 | 0.064 | 1.63 | 0.201 |
| LF Slope | 1.22 | 0.269 | 0.700 | 0.403 |
| Kappa | 2.30 | 0.130 | 2.79 | 0.095 |
| Mu | 0.191 | 0.662 | 1.91 | 0.167 |

**Table S5:** Summary of intra-sonographer repeatability results based on the first acquisition only during each examination.

| Measurement | Mean | Bias | Lower  LoA | Upper  LoA | SD | RC | CoV (%) | ICC |
| --- | --- | --- | --- | --- | --- | --- | --- | --- |
| AC  (dB/cm-MHz) | 1.2 | -0.023 | -0.50 | 0.45 | 0.24 | 0.47 | 14.5 | 0.46 |
| LF Intercept (dB) | -32.5 | -0.41 | -8.0 | 7.1 | 3.8 | 7.5 | 8.4 | 0.17 |
| LF Midband (dB) | -14.9 | -0.75 | -8.7 | 7.2 | 4.1 | 8.0 | 19.4 | 0.48 |
| LF Slope (dB/MHz) | 3.4 | -0.065 | -2.1 | 1.9 | 1.0 | 2.0 | 21.4 | 0.16 |
| Kappa | 0.50 | 0.007 | -0.076 | 0.089 | 0.042 | 0.082 | 5.9 | 0.56 |
| Mu | 3.2 | -0.18 | -1.5 | 1.2 | 0.69 | 1.3 | 15.1 | 0.50 |

**Table S6:** Summary of inter-sonographer reproducibility results based on the first acquisition only during each examination.

| Measurement | Mean | Bias | Lower  LoA | Upper  LoA | SD | RDC | CoV (%) | ICC |
| --- | --- | --- | --- | --- | --- | --- | --- | --- |
| AC  (dB/cm-MHz) | 1.1 | 0.024 | -0.48 | 0.53 | 0.26 | 0.50 | 15.8 | 0.30 |
| LF Intercept (dB) | -32.4 | -0.33 | -9.8 | 9.1 | 4.8 | 9.4 | 10.5 | 0.36 |
| LF Midband (dB) | -16.1 | 0.38 | -8.0 | 8.8 | 4.3 | 8.4 | 18.8 | 0.37 |
| LF Slope (dB/MHz) | 3.1 | 0.14 | -2.2 | 2.4 | 1.2 | 2.3 | 26.5 | 0.13 |
| Kappa | 0.49 | 0.011 | -0.12 | 0.14 | 0.065 | 0.13 | 9.3 | 0.12 |
| Mu | 3.2 | 0.091 | -1.5 | 1.7 | 0.82 | 1.6 | 17.9 | 0.33 |

**Fig. S1.** Repeatability Bland-Altman plots based on first acquisition only for (a) AC (dB/cm-MHz), (b) LF Intercept (LFI in dB), (c) LF Midband (LFM in dB), (d) LF Slope (LFS in dB/MHz), (e) Kappa (unitless), and (f) Mu (unitless). Dashed lines show bias and lower and upper LoA (95%); numerical values for bias and lower and upper LoA are displayed below each plot.


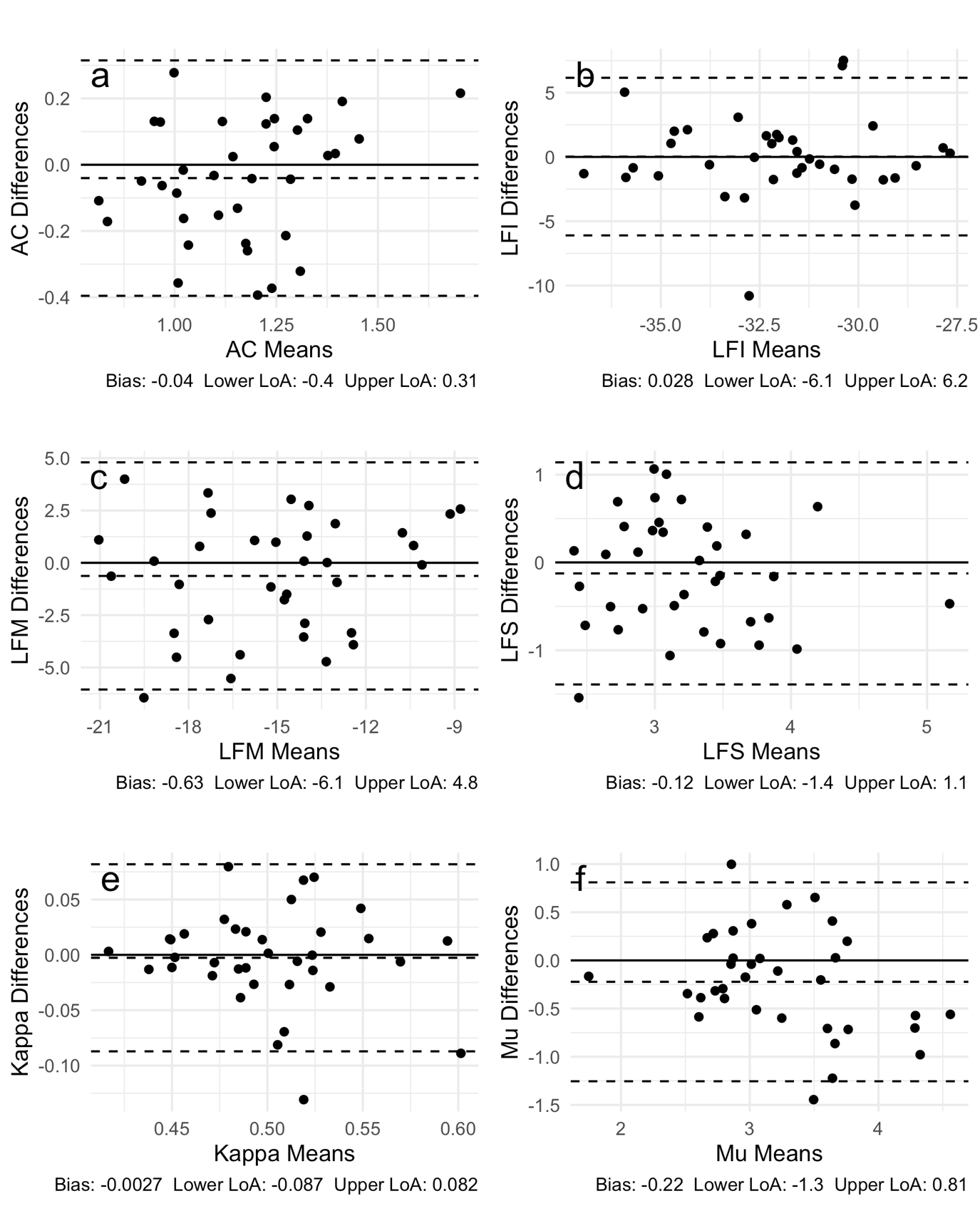


**Fig. S2** Reproducibility Bland-Altman plots based on first acquisition only for (a) AC (dB/cm-MHz), (b) LF Intercept (LFI in dB), (c) LF Midband (LFM in dB), (d) LF Slope (LFS in dB/MHz), (e) Kappa (unitless), and (f) Mu (unitless). Dashed lines show bias and lower and upper LoA (95%); numerical values for bias and lower and upper LoA are displayed below each plot.


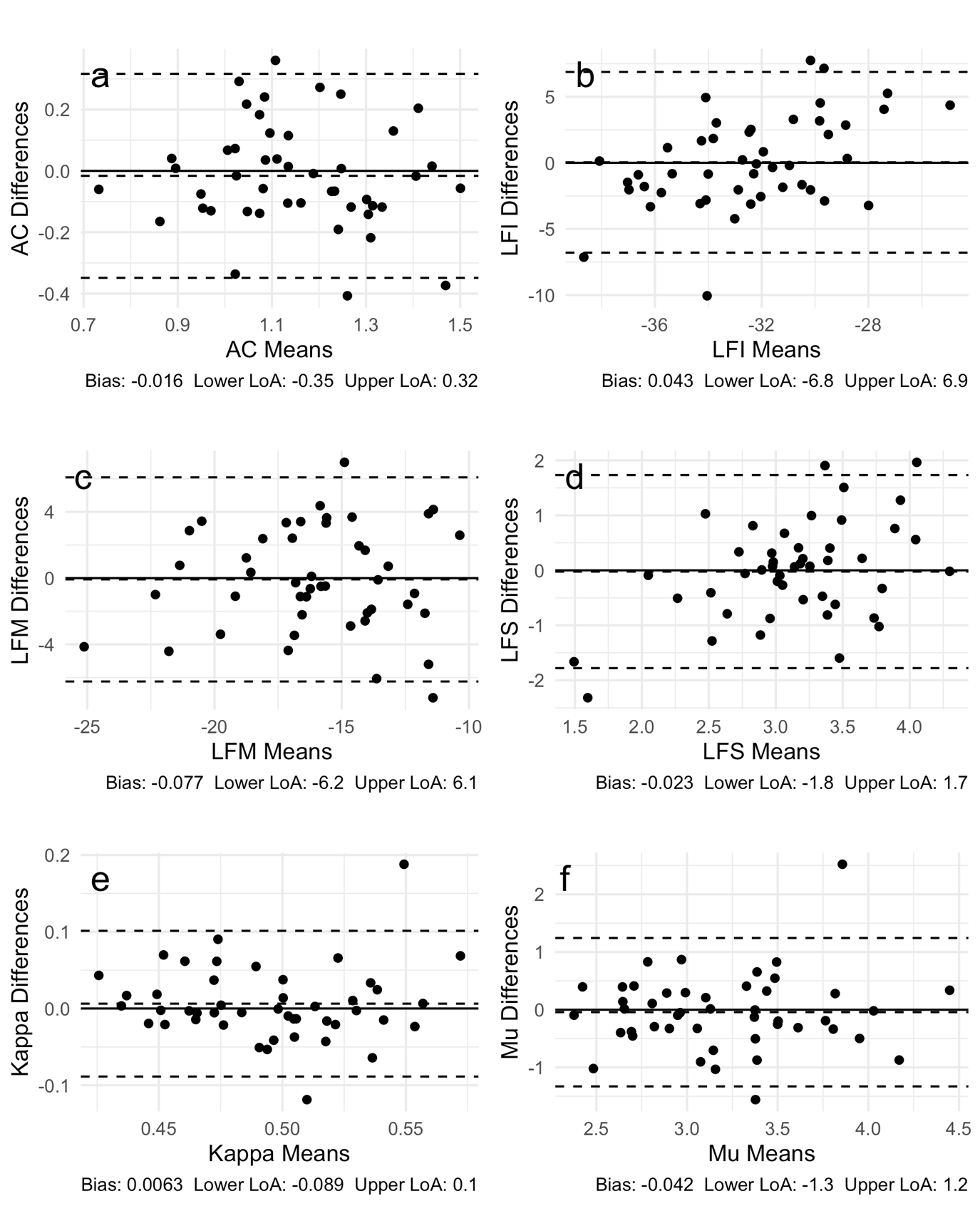


Data description

Supplementary file: Data_QUS.csv is a comma-separated variables file of the quantitative ultrasound biomarker data that were analyzed for repeatability and reproducibility after averaging over the first k acquisitions (k=1, 2, …, 10).

Variables are:

"Participant_ID" - random unique label

"Visit_ID" – indicates first (V1) or second (V2) research visit

"Transducer" – indicates which transducer was used

"Sonographer" – random unique label for sonographer

"Repeat_ID" – 1 indicates first exam by sonographer, 2 indicates second exam by sonographer during research visit

"Organ_scanned" – all are cervix

"Acquisition_index" – indicates repeat acquisitions 1 – 10 during exam

"Participant_scan_probe_cover" – LC or NLC cover type

"Phantom_scan_probe_cover" – NC indicates no cover used

"Phantom_used" – which of three phantoms were used for calibration

"FOI_analyst" – sonographer who set the field of interest

"Bandwidth_min_freq_in_MHz"

"Bandwidth_max_freq_in_MHz"

"Center_freq_in_MHz"

"AC_at_center_freq_in_dB_per_cm_MHz" – attenuation coefficient (AC)

"AC_number_of_subROIs" – number of AC subregions averaged

"BSC_LF_slope_in_dB_per_MHz" – Lizzi-Feleppa slope measurement

"BSC_LF_intercept_in_dB" - Lizzi-Feleppa intercept measurement

"BSC_LF_midband_fit_in_dB" – Lizzi-Feleppa midband measurement "BSC_at_center_freq_in_1_per_cm_sr" – backscatter coefficient, equivalent to LF midband but in original units instead of decibels

"BSC_number_of_subROIs" – number of subregions averaged for the BSC_LF measurements

"Envelope_stat_k_mean" – mean of kappa values over subregions

"Envelope_stat_k_std" – standard deviation of kappa values over subregions

"Envelope_stat_mu_mean" – mean of mu values over subregions

"Envelope_stat_mu_std" – standard deviation of mu values over subregions

Same-sonographer examinations share the same Participent_ID, Visit_ID, and Sonographer label, with Repeat_ID = 1 for the first exam and Repeat_ID=2 for the second exam.

Cross-sonographer examinations share the same and Participant_ID, Visit_ID, but have different Sonographer labels. In this case, Repeat_ID = 1 for both exams, as each is the first exam for the indicated sonographer during the visit.
